# Urinary BLACAT1 as a non-invasive biomarker for bladder cancer

**DOI:** 10.1007/s11033-023-08370-z

**Published:** 2023-03-20

**Authors:** Fathia Z. El Sharkawi, Mahmoud El Sabah, Hanaa B. Atya, Hussein M. Khaled

**Affiliations:** 1 Biochemistry and Molecular Biology Department, Faculty of Pharmacy, Helwan University, P.O. Box 11795, Cairo, Egypt; 2 Department of Biochemistry, Faculty of Pharmaceutical Sciences and Pharmaceutical Industries, Future University, Cairo, Egypt; 3 National Cancer Institute, Cairo University, Cairo, Egypt

**Keywords:** *BLACAT1*, Bladder cancer, *LncRNA*, Metastatic marker

## Abstract

**Background:**

Bladder cancer (BC) is recorded as the fifth most common cancer worldwide with high morbidity and mortality. The most urgent problem in BCs is the high recurrence rate as two-thirds of non-muscle-invasive bladder cancer (NMIBC) will develop into muscle-invasive bladder cancer (MIBC), which retains a feature of rapid progress and metastasis. In addition, only a limited number of biomarkers are available for diagnosing BC compared to other cancers. Hence, finding sensitive and specific biomarkers for predicting the diagnosis and prognosis of patients with BC is critically needed. Therefore, this study aimed to determine the expression and clinical significance of urinary *lncRNA BLACAT1* as a non-invasively diagnostic and prognostic biomarker to detect and differentiate BCs stages.

**Methods and results:**

The expression levels of urinary *BLACAT1* were detected by qRT-PCR assay in seventy (70) BC patients with different TNM grades (T0-T3) and twelve (12) healthy subjects as control. *BLACAT1* was downregulated in superficial stages (T0 = 0.09 ± 0.02 and T1 = 0.5 ± 0.1) compared to healthy control. Furthermore, in the invasive stages, its levels started to elevate in the T2 stage (1.2 ± 0. 2), and higher levels were detected in the T3 stage with a mean value of (5.2 ± 0.6). This elevation was positively correlated with disease progression. Therefore, *BLACAT1* can differentiate between metastatic and non-metastatic stages of BCs. Furthermore, its predictive values are not like to be influenced by schistosomal infection.

**Conclusions:**

Upregulation of *BLACAT1* in invasive stages predicted an unfavorable prognosis for patients with BCs, as it contributes to the migration and metastasis of BCs. Therefore, we can conclude that urinary *BLACAT1* may be considered a non-invasive promising metastatic biomarker for BCs.

## Introduction

Malignant bladder cancers are the greatest common tumors in the genitourinary system. Globally, Bladder cancer (BC) alarms around 550,000 new cases annually and its frequency is continually increasing [1]. BC is divided into two subgroups: non-muscle invasive bladder cancer (NMIBC), an early stage of cancer (stages T0 to T1), and muscle-invasive bladder cancer (MIBC), which is more destructive (stages T2 to T4). At diagnosis, the majority of BCs are NMIBC [2, 3]. Early diagnosis and handling of tumorous lesions are assumed to be essential for dropping the risk of relapse and improving the prognosis of NMIBC [4]. Regardless of enhancements in current clinical management such as surgery, radiation therapy, and chemotherapy, 50–70% of patients are relapsed within the next 5 years [5]. Hence, it is crucial to discover novel molecular markers for diagnosis at the primary stage and detect effective therapeutic marks for improving the survival rate of BC patients.

Long non-coding RNAs (*lncRNAs*) are a subgroup of RNAs that have above 200 nucleotides in length with no obvious open reading frames (ORFs); however, they have conventional secondary structures [6]. *LncRNAs* can interact with DNA, RNA, or proteins as molecular sponges, scaffolds, and activators to play key regulatory roles in a diversity of biological processes, such as proliferation, migration, invasion, differentiation, and apoptosis [7]. The role of *lncRNAs* in the manifestation and expansion of numerous human cancers has been widely studied and certain *lncRNAs* were described to act as promising markers for prognosis, estimation, or diagnosis for some cancer grades [8, 9].

With regards to BC, several studies have submitted that urine-based *lncRNAs* may function as talented biomarkers and may influence overall patient survival and mortality [10, 11]. Only a few *lncRNAs* have been proved experimentally, but their effects in regulating gene expression rest to be decoded.

Bladder cancer-associated transcript 1 (*BLACAT1*), also entitled linc-UBC1, is found at the locus of human chromosome 1q32.1 with a length of 2616 kb and has only 1 exon [12]. Initially, it was recognized by He et al. in 2013 and defined as non-coding based on sequence analysis [12]. Furthermore, nuclear fractionation of BC cells revealed that *BLACAT1* is preferentially located in the nucleus. Behind these results, *BLACAT1* engrossed a large amount of attention from cancer scientists. Many evolving studies revealed that *BLACAT1* was abnormally expressed in different cancers, including colorectal cancer (CRC) [13], gastric cancer (GC) [14], and lung cancer (LC) [15]. Furthermore, a meta-analysis showed by Lu et al. demonstrated that elevated *BLACAT1* expression could predict shorter survival, progressive TNM stage, and elevated lymph node metastasis in solid tumors [16]. These pioneer recommendations exhibited the clinical importance of *BLACAT1* in the diagnosis and prognosis of tumors.

Although, Urine Cystoscopy is accepted as a gold standard for BC screening, as it is non-invasive, low-priced, and safe. Even though it is highly specific, the results are not reproducible, and the explanation is extremely dependent on the cytologist’s skills [17]. This calls for exploring other molecular urine markers for BC screening which should be non-invasive, specific, sensitive, reproducible, and done at an appropriate cost. So, the present study aimed to decode our knowledge of the prognostic and diagnostic value of urinary *BLACAT1* for differentiating pathological grades of BCs in Egyptian patients.

## Patients and methods

The current study was achieved on seventy (70) BC Egyptian patients from the cystoscopy unit of the National Cancer Institute, Cairo University, in addition to twelve (12) healthy subjects as control. Demographic data and medical history were collected from patients’ hospital files. Tumor staging and grading were diagnosed according to the tumor, necrosis, and metastasis (TNM) classification of UICC (Union for International Cancer Control) [18]. Patients were classified according to their tumor grades into 4 groups with grades (T0–T3). Also, patients were further classified into the Schistosomal BC group (no = 37) and non-Schistosomal BC group (no = 33) as shown in (Table 2).

All subjects (patients and control) retained informed written consent for contribution and the study was approved by the research ethics committee for clinical studies at the faculty of pharmacy, Future University, Cairo, Egypt (No. of protocol: REC-FPSPI-2/17). All patients underwent cystoscopy as a standard reference for the identification of BC. The 12 healthy subjects served as control, were matched in sex and age with the BC group, and also, have no former history of any urological disorders.

### Samples collection and preparation

Fresh urine samples were collected from each subject in sterile containers (cups) and centrifuged in sterile Wassermann tubes within one hour of collection at 16,000 rpm for 15 min at room temperature to remove cellular material and debris from samples. Clear supernatants were separated in sterile Wassermann tubes under laminar flow with a HEPA filter and stored at -80 Cº until analysis. Patient samples were taken in the morning at the time of diagnosis before receiving any medication.

### RNA isolation and quantification

Total RNAs from urine supernatants were extracted using TRIzol® reagent (QIAamp Mini column, Qiagen, USA) according to manufacturer instructions. The concentration of the extracted RNA was measured using NanoDrop spectrophotometer.

### Determination of urinary levels of *BLACAT1* using quantitative PCR technique

Total extracted RNA was reversely transcribed to cDNA using a PrimeScript RT kit (Perfect Real Time, Takara, Holdings Inc., Kyoto, Japan) according to the manufacturer's protocol. BLACAT1 expression levels were performed by Real-time PCR assay technique using the following kits: TaqMan™ Non-coding RNA Assay kit, Catalog number: 4426961 provided by Applied Biosystems™ and master mix (TaqMan™ Fast Advanced Master Mix, Catalog number: 4444556, provided by Applied Biosystems™). The reaction volume used was 25 μL. Rotor gene-Q PCR machine (QIAGEN) detection system was used.

The thermo-cycling protocol was recorded as follows: First denaturation at 95˚C for 2 min, followed by 35 repeats of the three-step cycling program consisting of 30 s at 95˚C (denaturation), 1 min at 53˚C (primer annealing), and 30 s at 72˚C (elongation), followed by a final extension step for 10 min at 72˚C. The primer sequences used for qPCR are listed in (Table 1). The housekeeping gene GAPDH was used as the internal control. All reactions were performed in triplicate. The relative quantification of *BLACAT1* was normalized to GAPDH and determined based on the cycle threshold (CT) level as reported previously [19].

$$\Delta {\text{CT}}\; = {\text{ CT}}\;{\text{assessed}}\;{\text{gene - CT}}\;{\text{reference}}\;{\text{gene}}\;({\text{GAPDH}})$$

$$\Delta \Delta {\text{CT }} = \, \Delta {\text{CT }}\left( {\text{BC sample}} \right)\; - \;\Delta {\text{CT }}\left( {\text{healthy sample}} \right)$$

$${\text{RQ }}\left( {\text{Relative Quantification}} \right) \, = { 2}^{{ - (\Delta \Delta {\text{ct}})}}$$

**Table 1:** Sequences of primers for *BLACAT1* and housekeeping gene

Gene	Sequences
BLACAT1	Sense: 5'-GTC TCT GCC CTT TTG AGC CT-3' Antisense: 5'-GTG GCT GCA GTG TCA TAC CT-3'
GAPDH	Sense: 5'-GGG AAA CTG TGG CGT GAT-3' Antisense: 5'-GAG TGG GTG TCG CTG TTGA-3'

*BLACAT1* bladder cancer-associated transcript 1, *GAPDH* glyceraldehyde 3-phosphate dehydrogenase

### Statistical analysis

GraphPad Prism software was used to make the analyses (version 5.0). Quantification of data is represented as mean ± SEM. Student's t-test and one-way ANOVA were applied to estimate the statistical differences among different groups, where P < 0.05 was considered significant. The receiver operating characteristic (ROC) curve was used to differentiate between BC grades using MedCala 9.3.9.0 (MedCala, Mariakerke, Belgium).

## Results

### Demographic data

All clinical and demographic data of patients and control are shown in (Table 2). It was observed that age was not significantly different between groups (P > 0.05). The mean age of BC patients at the time of diagnosis was 61.3 years. While healthy controls had a mean age of 50.5 years.

**Table 2:** Demographic and clinical data of the studied subjects

	Schistosomal group	Non-schistosomal group	Total	Controls
Age (Mean):	62.77	59.8	61.3	50.5
Sex: Male Female	33 3	19 15	52 18	7 5
TMN stages: T0	8	4	12	
T1	7	7	14	
T2	13	11	24	
T3	9	11	20	
NMIBC (T0 + T1)	15	11	26	
MIBC (T2 + T3)	22	22	44	
Total	37	33	70	12

*TMN* tumor, necrosis, and metastasis, *NMIBC* non-muscle invasive bladder cancer, *MIBC* Muscle invasive bladder cancer

### Urinary levels of BLACAT1 in primary and invasive stages:

**Fig. 1:**
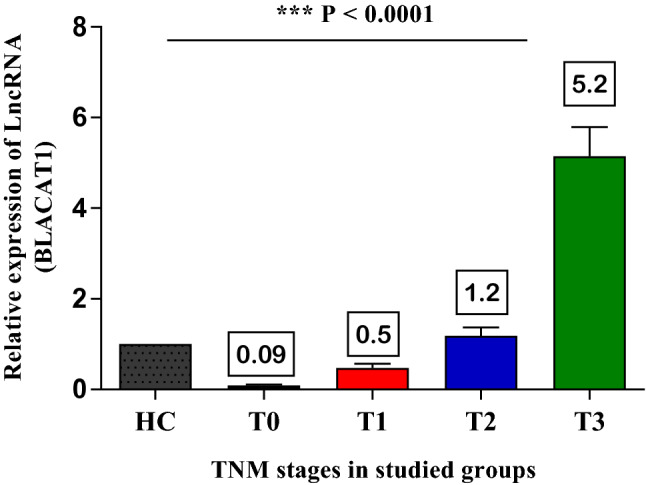
The relative expression levels of urinary *BLACAT1* in BC Patients. The values were expressed as mean ± SEM. ***P < 0.0001using ANOVA test

*BLACAT1* was downregulated in superficial stages (T0 = 0.09 ± 0.02 and T1 = 0.5 ± 0.1) compared to healthy control. However, in invasive stages, its levels started to elevate in the T2 stage (1.2 ± 0. 2), and higher urinary levels were detected in the T3 stage with a mean value of 5.2 ± 0.6. This elevation was positively correlated with disease progression (Fig. 1) & (Table 3).

**Table 3:** Urinary levels of *BLACAT1* in bladder cancer patients

Groups	Sample size (number)	Mean ± SEM
Healthy control (HC)	12	1
T0	12	0.09 ± 0.02
T1	14	0.5 ± 0.1
T2	24	1.2 ± 0. 2
T3	20	5.2 ± 0.6

*SEM* standard error of the mean

### Urinary levels of BLACAT1 in schistosomal and non-schistosomal BCs

**Fig. 2:**
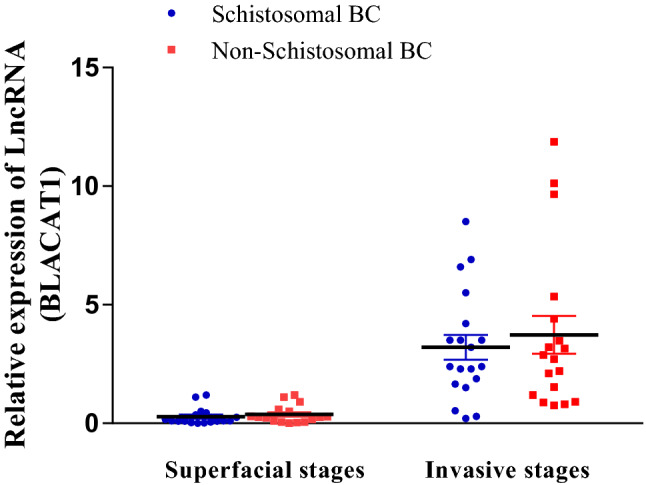
The relative expression levels of urinary *BLACAT1* in schistosomal and non-schistosomal BCs. P > 0.05 using unpaired t-test

When comparing the urinary levels of *BLACAT1* in schistosomal (37 cases) and non-schistosomal BC groups (33 cases); there was no significant difference between the two groups, where the superficial stages (T0 + T1) in schistosomal BCs showed expression levels of *BLACAT1* (0.28 ± 0.08) compared to (0.38 ± 0.1) in non-schistosomal BCs. While the invasive stages (T2 + T3) were (3.2 ± 0.52) in schistosomal BCs compared to (3.7 ± 0.8) in non-schistosomal ones, as shown in (Fig. 2).

### *Urinary levels of BLACAT1 in NMIBC (T0* + *T1) and MIBC (T2* + *T3):*

**Fig. 3:**
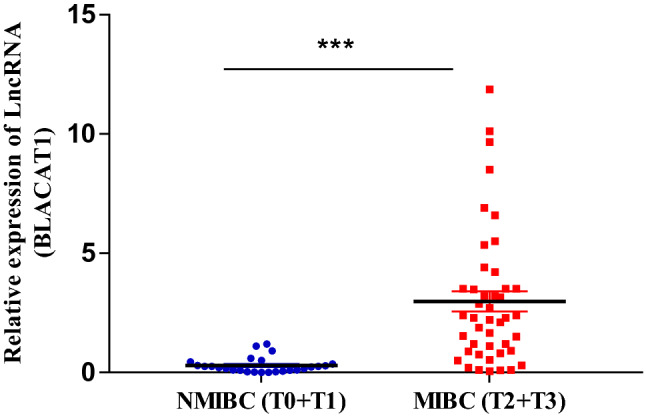
The relative expression levels of urinary *BLACAT1* in non-muscle invasive bladder cancer (NMIBC) and muscle-invasive bladder cancer (MIBC) groups. ***P < 0.0001using unpaired t-test

We evaluated the urinary levels of *BLACAT1* in a NMIBC group (T0 + T1, no = 26) and MIBC group (T2 + T3, no = 44) group (Fig. 3), and found that, *BLACAT1* demonstrated high expression levels in the muscle-invasive (MIBC) group (2.98 ± 0.43) compared to non- muscle-invasive (NMIBC) group (0.3 ± 0.06) at p < 0.0001.

### Diagnostic performance of BLACAT1 in bladder cancer detection:

**Fig. 4:**
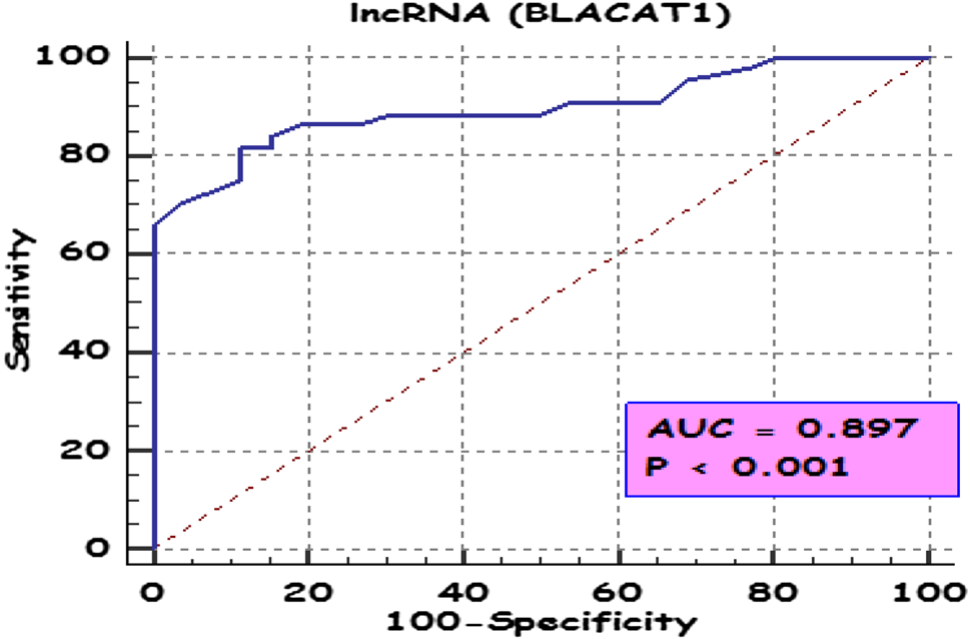
ROC curve of *BLACAT1* to differentiate between non-muscle invasive bladder cancer (NMIBC) and muscle-invasive bladder cancer (MIBC) in the studied groups

ROC analysis was performed to evaluate the sensitivity and specificity of *BLACAT1* as a diagnostic marker in BC patients, the area under the curve (AUC) was 0.52 with very high specificity (100%) and low sensitivity (51.4%). Furthermore, to evaluate the power of differentiating NMIBC patients from MIBC patients, the ROC curve was calculated (Fig. 4). *BLACAT1* demonstrated AUC values higher than 0.89 (p < 0.001) with overall sensitivity and specificity of 81.82 and 88.46% respectively, indicating higher performance to differentiate NMIBC patients from MIBC patients.

## Discussion

Bladder cancer (BC) is among the most fatal types of cancers worldwide [1]. Its aggressive type (MIBC) accounts for around 25% of all primarily diagnosed BC cases and 50% of patients die from metastatic disease even with the best therapeutic option [20, 21]. *lncRNAs* are a new class of gene managers in many cancer types [22]. They play important roles in an extensive range of biological processes among cancerous cells and might be involved in oncogenesis and tumor suppression [6, 23]. Additionally, lncRNAs also regulate the sensitivity of chemotherapy and radiotherapy of cancer cells [24].

*BLACAT1*, is branded as a new budding star, for a valuable and potential prognostic prediction for cancers [15, 16]. Our study revealed the *BLACAT1*upregulation of urinary *BLACAT1* in BC patients with higher TNM staging. Previous studies detected the upregulation of *BLACAT1* in BC tissues by more than1.5-fold, and this was associated with cell proliferation and migration in three BC cell lines (UMUC-3, TCCSUP, and 5637) [12, 25].

Furthermore, elevated expression of *BLACAT1* was associated with metastasis and higher tumor staging in breast cancer tissues and its downregulation decreased cell migration in SKBR3 and MDA-MB-231 cells with miR-150-5p overexpression [26]. Also, Depletion suppresses cell metastasis in ME180 and C33A cells were explored with Trans-well assay and wound-healing analysis [27].

The site of *lncRNAs* inside the cells often revealed their functions [25]. *BLACAT1* was enriched in the nucleus [22], it collaborated with the enhancer of zeste 2 (EZH2) and suppressor of zeste 12 (SUZ12), which were important modules of polycomb repressive complex 2 (PRC2), to modify the expression of some target genes [25]. Furthermore, the chromatin immunoprecipitation assay showed that *BLACAT1* could modify histone H3-lysine 27 (H3K27) tri-methylation level in the promoter regions of some genes, revealing its oncogenic role in BCs [12].

Our study is considered to be unique in the detection of *BLACAT1* expression levels in urine samples as non-invasive samples of BC patients. Numerous studies reported the upregulation of BLACAT1 in the tissue of many types of cancer, such as hepatocellular carcinoma, colorectal cancer, glioma, and osteosarcoma. Conversely, lower expression of *BLACAT1* was identified in normal tissues and the patient sera [24, 25].

Moreover, elevated *BLACAT1* expression was positively correlated with higher TNM staging, and patients with high *BLACAT1* expression were inclined to have a shorter overall survival [16]. Depletion of *BLACAT1* negatively affected cell proliferation, invasion, and migration in cervical cancer ME180 and C33A cells [27]. Likewise, Droop et al. also reported elevated *BLACAT1* expression in cancer tissues than in adjacent normal tissues, and this up-regulation was significantly related to poor survival in the cohort of The Cancer Genome Atlas (TCGA) BCs [28]. On the other hand, there was no apparent difference detected in the *BLACAT1* expression between tumor and benign tissues, and no significant association was found between metastasis and survival [25, 28]. This variation might be due to heterogeneity in patient populations, or difference in the detection techniques used. So, our study is considered to be a valuable unique one that detects a difference in urinary levels between patients and healthy people.

The present study was also unique in detecting *BLACAT1* expression in schistosomal BC patients although there was no significant variation in the levels of *BLACAT1* between schistosomal and non- schistosomal BC patients. Controversy, Hammam et al. determined the expression of the fibroblast growth factor receptor (FGFR3) gene in Egyptian BC patients and found that FGFR3 was significantly associated with schistosomal BC tumor grade and stage [29].

Our results demonstrated up-regulation of urinary *BLACAT1* in invasive BC stages (T2 + T3). This agrees with the results from Gao et al. who reported increased expression of *BLACAT1* in colorectal cancer tissues and this expression was more obviously expressed in CRC patients with greater tumor size, deeper tumor invasion, higher TNM stage, and more lymph node metastasis [13].

Different types of urinary BCs biomarkers were discussed before, such as the classical FDA-approved proteins, genetic and epigenetic biomarkers [30], and exosomal markers [30, 31].

When comparing our marker with urinary miRNAs measured in BCs patients, we found that *lncRNA BLACAT1* measured in the current study has higher sensitivity and specificity (81.82 and 88.46% respectively) as a diagnostic marker in the invasive stage (IMBC) while miRNAs included in the study of K.Ng et al., had high sensitivity and specificity (80% and more) in the non-invasive stage (NIMBC) [30]. So, urinary *lncRNA BLACAT1* in the current study may be considered a unique metastatic biomarker and can be used to differentiate between invasive and non-invasive bladder cancer stages. Furthermore, its predictive values are not like to be influenced by Schistosoma infection. Therefore, *lncRNA BLACAT1* is a promising prognostic biomarker for BCs.

In conclusion, we can conclude that *BLACAT1* may be considered one of the promising non-invasive metastatic biomarker for bladder cancer.

## Data Availability

All data generated or analyzed during this study are included in this published article.
